# Genomic Prediction for 25 Agronomic and Quality Traits in Alfalfa (*Medicago sativa*)

**DOI:** 10.3389/fpls.2018.01220

**Published:** 2018-08-20

**Authors:** Congjun Jia, Fuping Zhao, Xuemin Wang, Jianlin Han, Haiming Zhao, Guibo Liu, Zan Wang

**Affiliations:** ^1^Institute of Animal Science, Chinese Academy of Agricultural Sciences, Beijing, China; ^2^CAAS-ILRI Joint Laboratory on Livestock and Forage Genetic Resources, Institute of Animal Science, Chinese Academy of Agricultural Sciences, Beijing, China; ^3^International Livestock Research Institute (ILRI), Nairobi, Kenya; ^4^Institute of Dryland Farming, Hebei Academy of Agriculture and Forestry Sciences, Hengshui, China

**Keywords:** alfalfa, genomic prediction, agronomic trait, quality trait, estimated breeding value

## Abstract

Agronomic and quality traits in alfalfa are very important to forage industry. Genomic prediction (GP) based on genotyping-by-sequencing (GBS) data could shorten the breeding cycles and accelerate the genetic gains of these complex traits, if they display moderate to high prediction accuracies. The aim of this study was to investigate the predictive potentials of these traits in alfalfa. A total of 322 genotypes from 75 alfalfa accessions were used for GP of the agronomic and quality traits, which were related to yield and nutrition value, respectively, using BayesA, BayesB, and BayesCπ methods. Ten-fold cross validation was used to evaluate the accuracy of GP represented by the correlation between genomic estimated breeding value (GEBV) and estimated breeding value (EBV). The accuracies ranged from 0.0021 to 0.6485 for different traits. For each trait, three GP methods displayed similar prediction accuracies. Among 15 quality traits, mineral element Ca had a moderate and the highest prediction accuracy (0.34). NDF digestibility after 48 h (NDFD 48 h) and 30 h (NDFD 30 h) and mineral element Mg had prediction accuracies varying from 0.20 to 0.25. Other traits, for example, fat and crude protein, showed low prediction accuracies (0.05 to 0.19). Among 10 agronomic traits, however, some displayed relatively high prediction accuracies. Plant height (PH) in fall (FH) had the highest prediction accuracy (0.65), followed by flowering date (FD) and plant regrowth (PR) with accuracies at 0.52 and 0.51, respectively. Leaf to stem ratio (LS), plant branch (PB), and biomass yield (BY) reached to moderate prediction accuracies ranging from 0.25 to 0.32. Our results revealed that a few agronomic traits, such as FH, FD, and PR, had relatively high prediction accuracies, therefore it is feasible to apply genomic selection (GS) for these traits in alfalfa breeding programs. Because of the limitations of population size and density of SNP markers, several traits displayed low accuracies which could be improved by a bigger reference population, higher density of SNP markers, and more powerful statistic tools.

## Introduction

Alfalfa (*Medicago sativa* L) is the first most-important forage legume in the world, because of its high biomass yield (BY) and good nutritional quality. To meet the future demand of quantity and quality, the main objectives in alfalfa breeding programs are biomass related agronomic traits and nutrition value related quality traits. Though yield and quality of alfalfa have been improved by phenotypic selection, the genetic gain are relatively low compared to other crops, owing to many reasons, such as low heritability, complex genetic architecture, and high genotype-environment interaction (Annicchiarico et al., 2015a). Therefore, it is emergent that new breeding strategies should be introduced into alfalfa breeding programs to accelerate the genetic gain of targeted traits and thus to meet the increasing demands of forage production.

Breeding value (BV), known as genetic merit of an individual which cannot be measured directly, is always the key issue in plant breeding programs. However, accurately estimated breeding value (EBV) is impossible to be achieved in complex traits by using phenotypic data alone. To improve the accuracy of prediction, incorporating information of genetic markers, known as marker-assisted selection (MAS), is an optional strategy. The superiority of MAS than phenotypic selection is determined by the percentage of the genetic variance accounted for by the QTLs associated with the markers (Meuwissen and Goddard, 1996). Unfortunately, the proportion of variation in complex traits explained by significant markers is usually very small (Hayes and Goddard, 2010). Therefore, many markers in linkage disequilibrium (LD) with QTLs contributed to targeted traits are needed to realize a relatively high prediction accuracy.

Due to the decreased cost of high-throughput genotyping methods, huge amount of genomic information of many non-model plants has been produced. Utilization of genotypic information in plant breeding has become a highly prioritized research area in recent years. Since dense genetic markers covering whole genome are available in many species, a new method for estimating breeding value, namely the genomic selection (GS) or genomic prediction (GP), showed a great potential for enhancing the accuracy of GP of BV (Meuwissen et al., 2001). It is assumed that all genes, with either large or small effects, affecting targeted traits are in LD with some markers that are distributed across the genome, paving the way to achieve a high accuracy of genomic estimated breeding value (GEBV) (Meuwissen, 2007). In a simulation study, the accuracy could be as high as 0.85 (Meuwissen et al., 2001). But this is not always the case in the real data. Several studies on GP have been done in wheat (Lado et al., 2013; Jiang et al., 2017; Sukumaran et al., 2017), maize (Riedelsheimer et al., 2012; Crossa et al., 2013; Pace et al., 2015), and other plants (Shu et al., 2013; Xu et al., 2014; Grenier et al., 2015), revealing a majority of the prediction accuracies between 0.05 and 0.8, depending on the traits, statistical methods, and experiment designs.

As mentioned above, GP can significantly improve the accuracy of estimation of breeding value. Therefore, it attracts a great interest of plant breeders worldwide. Traits being targeted in plant breeding programs are either difficult or costly to be measured. Additionally, the targeted traits (e.g., yield, phenology, and adaptation to stress) in plant breeding are mostly quantitative traits, which are controlled by multiple genes and generally sensitive to environmental variables. Phenotypic selection, neglecting the underlying biological processes and the interactions between genes and environments, cannot make a significant genetic gain in a short time frame. Considering the genetic architecture of the quantitative traits, MAS is also not the best choice. GP, following its assumption, is thus an ideal tool to be used in the plant breeding programs. Many methods have been adopted for GP or GS. Bayesian methods and GBLUP, however, are those being frequently used. Bayesian methods exhibited more advances than GBLUP in terms of prediction accuracy following a simulation study (Meuwissen et al., 2001). No matter which method is used for GP, the density of markers across the whole genome is a determining factor. Typically, two types of high throughput genotyping methods of SNP array and whole-genome re-sequencing can be employed to generate high quality genotypes of markers. For important crop species, several SNP Bead chips at different marker densities have been developed (Ganal et al., 2012). Because of the lack of SNP array, genotyping by sequencing (GBS) is therefore an alternative to alfalfa genotyping. In the current study, we investigated the impact of three Bayes statistical methods on the prediction accuracies of alfalfa agronomic and quality traits with genotypic data obtained by GBS.

## Materials and Methods

### Plant Materials and Experimental Designs

The alfalfa materials used in this study were consisted of 322 genotypes representing 75 tetraploid alfalfa accessions under the experimental designs as described in Wang et al. (2016).

### Phenotypic Data Collection and Analysis

A total of 25 traits (**Table 1**), including 15 quality and 10 agronomic traits, were measured for all genotypes. All the plants were harvested at early flowering stage and prepared to measure the 15 quality traits using a FOSS 5000 scanning monochromator (FOSS, Denmark). The 15 quality traits included three fiber-related traits, four digestibility-related traits, and eight nutrition component traits being measured following the procedures described in our previous studies (Wang et al., 2016; Jia et al., 2017). Before harvesting, plant height (PH) of each plot was measured as nature height on every plant. Plant branch (PB) was measured as the number of primary branches arising from the main stem. The number of main stem node (SN) for each plot was directly counted since the first node on the main stem from every plant. The first inflorescence position (FP) was measured as the position of the first inflorescence on the stem. After harvesting, BY was measured as the fresh weight by clipping all six plants in each plot at a uniform height of 5 cm. The stems and leaves were separated and placed into a nylon net bag, naturally air-dried, and weighed separately to calculate the leaf to stem ratio (LS). Meanwhile, dry matter (DM) was defined as the sum of the weights of stems and leaves. Plant regrowth (PR) was measured as the PH two weeks after the first harvest. Flowering date (FD) was calculated by the date of opening of the first flower for the first two growth cycles. PH in fall (FH) was measured as the PH 21 days after the last harvest. The mean value of all six plants in each plot represents the trait value of a genotype grown in that plot. The measurements of all traits were performed on all genotypes under three consecutive years (2013, 2014, and 2015).

**Table 1:** Prediction accuracies of 25 traits.

	Prediction accuracies		
Traits	BayesA	BayesB	BayesCπ
ADF	0.1847	0.1805	0.1824
aNDF	0.1843	0.1942	0.1958
dNDF30	0.2002	0.1933	0.1963
dNDF48	0.2538	0.2545	0.249
ADL	0.0783	0.0756	0.08
IVTDMD30h	0.0907	0.0906	0.0889
IVTDMD48h	0.0994	0.1029	0.1002
CP	0.0528	0.0524	0.0557
RUP	0.0828	0.0909	0.0746
Ash	0.087	0.0836	0.085
Ca	0.337	0.3422	0.3393
K	0.1663	0.1586	0.1572
Mg	0.2184	0.2183	0.2178
P	0.1178	0.1196	0.1203
Fat	0.1148	0.1203	0.1114
BY	0.2418	0.2511	0.2457
DM	0.1285	0.1257	0.1271
FD	0.5153	0.5139	0.5131
FH	0.6485	0.6466	0.6451
FP	0.0639	0.0596	0.0683
LS	0.3214	0.3215	0.3249
PB	0.2589	0.2598	0.2593
PH	0.1587	0.1601	0.1626
PR	0.5105	0.5111	0.5074
SN	0.0045	0.0047	0.0021

*Abbreviations of traits are explained in Materials and Methods.*

Linear mixed model was fitted to calculate the BLUP value and EBV for individual trait of each genotype as follows:

$${\text{y}}_{i}=\mu +{\text{g}}_{i}+{\text{e}}_{i}+\varepsilon_{i}.$$

In this equation, y*_i_* represents the phenotype of the *i*th genotype, μ is the grand mean value of the targeted trait in all environments, g*_i_* is denoted as genetic effect, e*_i_* is the environmental effect, and 𝜀*_i_* is the random error. The BLUP value was estimated for individual trait of each genotype based on the above-mentioned linear model using the lme4 model (Bates et al., 2011). The EBV of individual genotype was used as response value in GP equation to estimate marker effect.

### DNA Isolation, GBS Library Construction, Sequencing, and Genotypic SNP Calling

Leaf tissues were collected from all genotypes and DNAs were extracted using the Qiagen DNeasy 96 Plant kit (Qiagen, CA, United States). DNA degradation and contamination were monitored on 1% agarose gels. DNA purity and concentration were checked using the NanoPhotometer^®^ spectrophotometer (IMPLEN, CA, United States) and Qubit^®^ DNA Assay Kit in Qubit^®^ 2.0 Flurometer (Life Technologies, CA, United States), respectively. DNA was digested by *Mse*I [New England Biolabs (NEB)] restriction enzyme. The reduced representation libraries were constructed for individual genotypes according to published GBS protocol (Elshire et al., 2011) and sequenced using Illumina HiSeq2000 platform. Raw data were submitted to the NCBI Sequence Read Archive with a reference number of SRP081825. The Tassel 3.0 Universal Network Enabled Analysis Kit (UNEAK) pipeline (Lu et al., 2013) was used for *de novo* SNP discovery and genotype calling following Li et al. (2014).

### SNP Imputation

After SNP calling, NPUTE was used to impute the GBS data (Roberts et al., 2007).

### Statistical Methods for GP

Three regression methods with different prior assumptions of the distribution of marker effects were used to estimate SNP effects, namely the BayesA (Meuwissen et al., 2001), BayesB (Meuwissen et al., 2001), and BayesCπ (Habier et al., 2011). A ten-fold cross validation was used to evaluate the accuracy of GP. The data were randomly split into 10 approximately equal-sized groups. For each cross validation, nine groups were used as the training population to estimate parameters and the remaining group (validation population) was used as the test sample. The linear model is denoted as follows:

$$y_{i}=\mu +\sum_{j=1}^{m}Z_{ij}\alpha_{j}+e_{i}$$

where, *y_i_* is the EBV of one trait, μ is the overall mean, *m* is the number of markers, *Z_ij_* is the *jth* SNP genotype of plant *i*, α*_j_* is iistheresidualerrorwithanassumednormaldistributionN(*0,* σ*^2^_e_*). SNP effects were estimated based on the training population using this equation. The GEBV for plant *i* in the validation population was predicted by summing up SNP effects over all loci. Predictive accuracy was measured as the correlation between the EBVs and GEBVs. Random sampling training and validation sets were repeated 10 times and the mean of correlations was calculated to measure the GP accuracy. All Bayes programs were run in BGLR package in R environment.^1^ The number of Burn-in was 10000, thin was 20, and the total number of iteration was 30,000. Other priors of parameters were assigned following Perez and de los Campos (2014).

## Results

### Phenotypic Variation

Since our previous works have described the phenotypic variations of some fiber-related traits (Wang et al., 2016) and crude protein and mineral elements (Jia et al., 2017), we will not describe them in this study. Instead, we want to represent the EBV variations of all traits incorporated in this study. The frequency distributions of EBVs for all 25 traits were symmetric as shown in **Supplementary Figure S1**.

### GP Using Three Bayesian Methods

**FIGURE 1:**
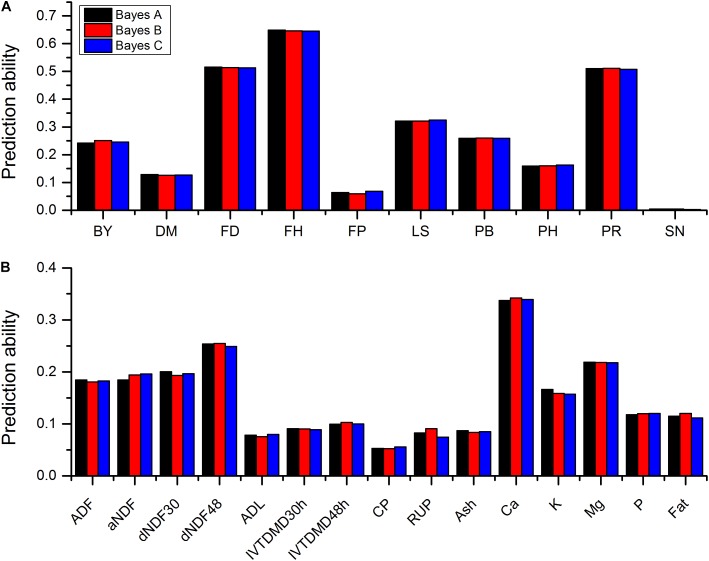
Predictabilities of 10 agronomic traits **(A)** and 15 quality traits **(B)** plotted against three Bayesian methods. Different colors represent different methods. Abbreviations of traits are explained in Materials and Methods.

Sequencing of the GBS libraries yielded approximately 184.59 million raw reads and 178.2 million clean reads in all 322 alfalfa genotypes. After imputation, 44,757 high quality SNPs were obtained and used for GP. The results of prediction accuracies of three Bayesian methods are shown in **Table 1**. The predictabilities drawn from the ten-fold cross validation varied across different traits. SN had the lowest predictability (0.0021) but FH had the highest predictability (0.6485). Some quality traits such as crude protein (CP), RUP, and ADL exhibited relatively low prediction accuracies (< 0.1) while the remaining quality traits such as fat, K, and Ca showed low to moderate predictabilities (0.11-0.34). Agronomic traits hold similar patterns except three traits that had relatively high predictabilities with FH to be the highest (0.65), followed by FD (0.52), and PR (0.51). Other traits, such as LS, PB, and BY displayed moderate predictabilities (0.24-0.32). Similar to BayesA method, BayesB and BayesCπ methods did not reveal any significant difference from each other in terms of the predictabilities of all quality and agronomic traits (**Table 1** and **Figure 1**). The predictabilities among the three Bayesian methods are shown in **Figure 1**. From the bar-plotting, only minor differences were observed among the three methods for all 25 traits, it was therefore hard to determine which method was the best.

## Discussion

Since GS was proposed by Meuwissen et al. (2001), many studies have been conducted in major crop species (Heffner et al., 2011a,b; Zhao et al., 2013; Iwata et al., 2015; Spindel et al., 2015) and farm animals (Fang et al., 2017; Hay and Roberts, 2017; Tan et al., 2017). The application of GPs to alfalfa BY and forage quality breeding were also initiated recently (Annicchiarico et al., 2015b; Li et al., 2015; Biazzi et al., 2017). In alfalfa industry, BY and forage quality are the key traits for genetic improvement. Other than the direct traits such as PH, BY, and DM that can inflect the BY of alfalfa, some phenology-related agronomic traits such as FH can also affect the BY. In this study, we therefore investigated the possibility of GP applied to alfalfa germplasm resources and GS applied to 10 important agronomic traits and 15 forage quality traits of alfalfa production.

Several methods, such as random regression BLUP, Bayesian methods and GBLUP, were employed to estimate GP and GS. Some simulation studies on different species suggested Bayesian methods to be superior than GBLUP in terms of the prediction accuracy (Meuwissen et al., 2001; Fernando et al., 2007; Clark et al., 2010; Zhang et al., 2010; Calus and Veerkamp, 2011). Compared with other methods, Bayesian methods also possessed other advantages (Gonzalez-Recio et al., 2010). In this study, we used the empirical data of 25 traits of 322 genotypes of 75 alfalfa accessions to compare the performance of GP following three statistical approaches of BayesA, BayesB, and BayesCπ. The BayesA method is based on the assumption that the prior distribution of variances of SNPs followed the scaled inverted chi-square distribution, implicating many SNPs with small effects and a small proportion of SNPs with moderate effects. BayesB assumes that many of the SNPs have no effect and the prior distribution of the variances of SNPs is a mixture of a distribution with zero variance and an inverse chi-squared distribution (Meuwissen et al., 2001). BayesCπ, however, treats the prior probability π that a SNP has zero effect as unknown (Habier et al., 2011). **Figure 1** shows that these three Bayesian methods demonstrated very similar prediction accuracies across all 25 traits, irrespective of their different assumptions. BayesA, BayesB, and BayesCπ identified six, five, and four quality traits as well as three, four, and three agronomic traits having the highest accuracies, respectively.

Besides the methods of GP discussed above, there are other factors affecting the prediction accuracies. One of them is the population composition and structure. Therefore, EBVs were directly used as the response variable to GP rather than phenotypes in the study. Since EBVs were corrected for non-genetic effects, it can be readily captured by SNPs using the Bayes methods. Methods of imputation for SNP genotypes are also important (Moghaddar et al., 2015).

Compared to previous studies, there were some differences in the accuracies of prediction for both agronomic and quality traits. For example, Biazzi et al. (2017) reported a very low accuracy (∼0.1) for LS which had nonetheless a moderate value at 0.32 in our study. DM showed a low accuracy (0.13) in our study, but Annicchiarico et al. (2015b) identified a moderate value of 0.35 in two genetically distinguished alfalfa populations. For BY, previous study showed moderate to high accuracies (0.21-0.66, Li et al., 2015) while it had an accuracy at 0.25 in the present study.

All the 15 quality traits had relatively low prediction accuracies due probably to their low heritabilities (Wang et al., 2016; Jia et al., 2017) determined by the genetic complexity of these traits. Biazzi et al. (2017) detected moderate prediction accuracy values for stem dNDF and leaf protein content (0.3–0.4) followed by leaf ADL and dNDF while the remaining traits showed low to very low accuracies. In our study, the accuracy of dNDF was almost moderate, similar to that of leaf dNDF but slightly lower than stem dNDF. These differences may be attributed to different sizes of reference populations, training populations, and number of markers. Different statistical models may lead to such discrepancies. The methods of imputation of SNP genotypes can also affect the accuracy of prediction (Moghaddar et al., 2015).

The present study was an attempt to predict alfalfa GEBVs of 25 important traits associated with BY and forage quality using three Bayesian statistical methods. Overall, they all exhibited similar predictabilities. Some traits possessed relatively high prediction accuracies (e.g., FH, FD, and PR with accuracies of 0.65, 0.52, and 0.51, respectively). Therefore, it is feasible to apply GS on these traits in alfalfa breeding programs. While GS/GP may be poorly effective for other traits such as ADL, crude protein, and RUP with low prediction accuracies.

## Author Contributions

ZW designed the experiments. HZ, XW, and GL phenotyped the traits. CJ and FZ analyzed the data and drafted the manuscript. ZW and JH revised the manuscript. All authors have read and approved the final manuscript.

## Conflict of Interest Statement

The authors declare that the research was conducted in the absence of any commercial or financial relationships that could be construed as a potential conflict of interest.

## Footnotes

1 http://www.r-project.org

## Supplementary Material

The Supplementary Material for this article can be found online at:

FIGURE S1 Distribution of EBVs for 25 traits. Abbreviations of traits are explained in Materials and Methods.
